# Should we hunt a metric?

**DOI:** 10.1093/jhps/hnz034

**Published:** 2019-07-29

**Authors:** Richard (Ricky) Villar

**Affiliations:** Editor-in-Chief, Journal of Hip Preservation Surgery

He is called Charles Goodhart and if you are an economist you should know the name well. Time was he was seriously influential in the UK’s Bank of England, especially during the era of Margaret Thatcher. His words have led to a statement, now known as Goodhart’s Law. ‘When a measure becomes a target’, it says, ‘it ceases to be a good measure’. His words may have been focussed on the world of economics, but a recent publication on the metrics of academic publishing, by Fire and Guestrin [1], more than grabbed my attention and has shown me how Goodhart’s Law applies just as much to academia. For me as an Editor, theirs was a paper I could not put down.

Once again, we are back to impact factor, that 60-year-old metric devised by Garfield [2], and which we all love to hate. Yet the fact is that academic publishing is changing, is described as being hypercompetitive, and an environment where it becomes increasingly difficult to maintain scientific integrity [3], as quantity becomes seemingly more important than the quality we seek.

The Fire and Guestrin study was truly astonishing. In it they analysed more than 120 million papers, with 528 million references, 35 million authors, more than 2600 research fields, since the start of the 19th century. Their conclusion was straightforward. They did not consider that citation-based metrics were helpful when comparing researchers in different fields, or for that matter in the same department. In our so-called modern era, where much of life is led head-down, staring at a touch-screen companion, there is an increasing trend to publish in preprint repositories [4]. One such site, bioRxiv reported that 1 million studies were downloaded each month, largely in neuroscience, bioinformatics and genomics [5]. Two-thirds of preprints posted before 2017 were later published in peer-reviewed journals. There was also a positive correlation between the number of downloads of a preprint and the impact factor of the journal in which it ended up [6]. The higher the number of downloads, the higher the impact factor of the journal.

There is a trend also to now publish in megajournals. These are journals where papers are reviewed for integrity, not always impact, and can have acceptance rates of >50%. An established megajournal, *PLoS One,* is no longer the largest in the world, a title now held by *Scientific Reports* (Springer Nature). *PLoS One* published 5541 articles in the first quarter of 2017, *Scientific Reports* published 6214 [7]. Game, set and match to the latter.

Hyperauthorship is becoming increasingly common [8–11], authors sometimes numbering in the thousands. Titles are changing to attract more online attention [12] and the descriptive term for this—academic clickbait [13, 14]—is the phrase to remember. Each of us wishes to attract others to our paper. All types of tactic can be used to achieve this. It is also commonly thought that these days we are publishing more than was once the case. It is true that the number of papers is climbing enormously. For example, a study by Herrmannova and Knoth [15], using the Microsoft Academic Graph, which displays a dataset of almost 115 million publications, reported that there were fewer than 1 million papers published in 1980. This figure climbed to more than 7 million in 2014.

However, the publication activities of researchers in the first 15 years of their careers, over more than a century, were looked at by Fanelli and Larivière [16], who concluded that if collaboration and co-authorship were taken into account, the publication rate of scientists in all disciplines has actually declined. They went on to decide that although we may believe that pressure to publish is causing the scientific literature to be flooded with salami-sliced, trivial, incomplete, duplicated, plagiarized and fabricated publications, this may well be incorrect. Are they right? Let us see.

As the world of scientific publishing changes, so traditional metrics come more under scrutiny. There is the impact factor, h-index, citation number, c-index, q-index, w-index, Scimago Journal Rank [17], altmetric and others. Yet the moment a metric appears, so it is open to manipulation. One can increase self-citations [18, 19], index false papers [20], merge papers on Google Scholar [21, 22] and more. Merging is quite commonly undertaken, and should that be your thing, the h-index is best manipulated by merging articles with widely dissimilar titles. The problem, of course, is that metric manipulation has almost become normalized [23]. The publishing we once understood is changing in mysterious ways.

So, what of the future? Definitely keep an eye out for pre-publication repositories, as these are becoming more dominant almost by the day. Think, too, if the impact factor is truly a metric that should trouble you, especially when publishing is changing so fast. Does Goodhart’s Law apply to this metric? I rather suspect it might. And as for this journal, our journal—*JHPS*—it is doing amazingly well. It seems only yesterday our first issue appeared, but here we are already with Volume 6. This is clearly thanks to a fantastic Editorial Board and a first-class cohort of reviewers and authors. *JHPS* keeps going while so many others fail.

Turning to the journal, our last issue, issue 6.1 was tremendous. I realize I am biased but look at the paper by Webb *et al.* [24] on capsular adhesions, in which the authors recommend preserving the chondrolabral junction at surgery as a way of reducing the chances of symptomatic adhesions developing post-operatively. The paper makes a strong case for preserving the junction and is tremendously helpful to the practising hip preservation specialist. Time was, all labra came off, rims were trimmed, and labra reattached. Now there is a choice.

My other hot favourite was the review written by Dallich *et al.* [25] on chondral lesions of the hip, and their anatomy, imaging and treatment. I learned much from it and was pleased to read about chondroplasty, microfracture, cartilage transplants and other orthobiological techniques. This review of chondral matters, pathology with which any hip preservation specialist must deal, was timely and well presented.

This issue, issue 6.2, is also filled with excellence. Once more it is impossible to choose between the papers, but two held my attention for slightly longer. The paper by Lemos *et al.* on the superior glutaeal vein syndrome was a real find. Intrapelvic neurovascular conflict should definitely be considered in cases of sciatica but with no identifiable musculoskeletal aetiology [26]. I am a glutton for anatomy, too, so much enjoyed the paper from Plante *et al.* [27] on the anatomical variants of rectus femoris motor innervation. I now know that I should not dissect medial to the rectus femoris any further than 7 cm distal to the anterior inferior iliac spine. That fact proved to be useful the other day when I was removing a mass of heterotopic ossification from the front of a hip joint, so thank you Plante *et al.* for your paper.

So, as ever, please enjoy this issue of *JHPS*. It is published for you, the hip preservation practitioner, and is filled from cover to cover with brilliance. I commend this issue to you in its entirety.

My very best wishes to you all.
